# Hippocampal avoidance with volumetric modulated arc therapy in melanoma brain metastases – the first Australian experience

**DOI:** 10.1186/1748-717X-8-62

**Published:** 2013-03-18

**Authors:** Raef Awad, Gerald Fogarty, Angela Hong, Patricia Kelly, Diana Ng, Daniel Santos, Lauren Haydu

**Affiliations:** 1 Genesis Cancer Care, Crows Nest, NSW, Australia; 2 Radiation Oncology Department, Mater Hospital, Crows Nest, NSW, Australia; 3 Melanoma Institute Australia, Sydney, NSW, Australia; 4 Central Clinical Schools, the University of Sydney, Sydney, NSW, Australia; 5 Discipline of Surgery, Sydney Medical School, the University of Sydney, Sydney, NSW, Australia

**Keywords:** VMAT, SIB, Brain metastases, Melanoma, Hippocampal avoidance

## Abstract

**Purpose:**

Volumetric modulated arc therapy (VMAT) can deliver intensity modulated radiotherapy (IMRT) like dose distributions in a short time; this allows the expansion of IMRT treatments to palliative situations like brain metastases (BMs). VMAT can deliver whole brain radiotherapy (WBRT) with hippocampal avoidance and a simultaneous integrated boost (SIB) to achieve stereotactic radiotherapy (SRT) for BMs. This study is an audit of our experience in the treatment of brain metastases with VMAT in our institution.

**Methods and materials:**

Metastases were volumetrically contoured on fused diagnostic gadolinium enhanced T1 weighted MRI/planning CT images. Risk organs included hippocampus, optic nerve, optic chiasm, eye, and brain stem. The hippocampi were contoured manually as one paired organ with assistance from a neuroradiologist. WBRT and SIB were integrated into a single plan.

**Results:**

Thirty patients with 73 BMs were treated between March 2010 and February 2012 with VMAT. Mean follow up time was 3.5 months. For 26 patients, BMs arose from primary melanoma and for the remaining four patients from non-small cell lung cancer (n= 2), primary breast cancer, and sarcoma. Mean age was 60 years. The male to female ratio was 2:1. Five patients were treated without hippocampal avoidance (HA) intent. The median WBRT dose was 31 Gy with a median SIB dose for BMs of 50 Gy, given over a median of 15 fractions. Mean values for BMs were as follows: GTV = 6.9 cc, PTV = 13.3 cc, conformity index = 8.6, homogeneity index = 1.06. Mean and maximum hippocampus dose was 20.4 Gy, and 32.4 Gy, respectively, in patients treated with HA intent. Mean VMAT treatment time from beam on to beam off for one fraction was 3.43 minutes, which compared to WBRT time of 1.3 minutes. Twenty out of 25 assessable lesions at the time of analysis were controlled. Treatment was well tolerated; grade 4 toxicity was reported in one patient. The median overall survival was 9.40 months

**Conclusions:**

VMAT for BMs is feasible, safe and associated with a similar survival times and toxicities to conventional SRT+/−WBRT. The advantage of VMAT is that WBRT and SRT can be delivered at the same time on one machine.

## Introduction

Brain metastases (BMs) are common in metastatic melanoma and are associated with a decrease in quality and quantity of life [1-3]. Melanoma cerebral metastases tend to be multiple [4,5], and the cause of death in most patients is central nervous system disease rather than extra- cranial systemic disease [4,6,7]. Steroid therapy, neurosurgery, stereotactic radiotherapy techniques (SRT), and Whole Brain Radiation Therapy (WBRT) with or without SRT have been the standard of care [8]. Currently, patients having SRT and WBRT require two separate courses of radiotherapy and two different plans, and usually on two separate machines or even perhaps separate centres. Conventional SRT can consume significant linear accelerator time. WBRT has been associated with neurocognitive sequelae [9-12]. The organ at risk is thought to be the limbic system especially the hippocampus [13,14].

Volumetric modulated arc therapy (VMAT) can deliver conformal dose distributions like those delivered with intensity modulated radiotherapy (IMRT) accurately, but the delivery times are much faster than conventional IMRT. The recent introduction of VMAT has therefore allowed the expansion of conformal techniques to more cases including palliative scenarios [15]. VMAT has enabled WBRT with hippocampal avoidance (WBRTHA), with simultaneous Integrated boost (SIB) to regions of macroscopic tumour, all within the one radiotherapy course. As both the WBRT and SIB doses are delivered simultaneously and within the same plan, there are advantages in terms of better dosimetry, radiobiological advantage and quality assurance compared to sequential WBRT and radiosurgery (SRS) boosts, which are delivered in separate courses at different times, often on different machines [16,17].

Currently the Radiation Therapy Oncology Group (RTOG) is investigating whether WBRTHA preserves neurocognitive function (NCF) in patients with BMs using IMRT techniques that include VMAT. In order to fulfil the credentialing and attain in-house practice for this trial, we planned and treated patients with BMs predominantly from melanoma. This is the first reported Australian experience of this technique with patients treated with WBRT with either hippocampal avoidance (HA), SIB, or both.

## Methods and materials

Patients treated with this technique were analysed retrospectively after approval by Sydney south west area health service (SSWAHS) Ethics Review Committee. No formal testing of hippocampal quality of life was performed either before or after treatment in these patients. Patients were treated with a Varian 21IX machine (RapidArc, Varian medical systems] using two complementary arcs. Planning software was Eclipse radiotherapy treatment planning system, version 8.6.07^®^™ (Varian Medical Systems Inc.). A planning target volume (PTV) for the whole brain was derived from auto-segmentation of the brain on the CT scan, with the addition of a 2 mm symmetrical margin. For patients treated with SIB to individual metastases, the gross tumour volume (GTV) were volumetrically contoured on fused diagnostic gadolinium enhanced T1 weighted MRI/planning CT images. GTV for each target metastasis was identified as the gadolinium -enhancing lesion on the T1- weighted MRI. PTV for each metastasis was outlined using a computer-automated 2 mm 3D margin expansion of the GTV for each metastasis. Organs at risk included hippocampus, optic nerve; optic chiasm, eye, and brain stem were outlined.

### Method of hippocampal avoidance

Both hippocami were defined as one paired organ. The hippocampi were contoured manually with assistance from a neuroradiologist, with reference to the on-line RTOG contouring atlas specified on (http://www.rtog.org/corelab/contouringatlases/hippocampalsparing.aspx). As per ongoing RTOG 0933 protocol, HA regions were generated by three-dimensionally expanding the hippocampal contours by 5 mm. The HA regions were subtracted from the WBRT PTV. WBRT and SIB were integrated into a single plan. Both WBRT and SIB doses were prescribed at 100%, according to the ICRU criteria [18]. The minimally accepted doses to the WBRT PTV and the SIB PTV were 95% of the prescription dose. There was no maximum dose limit for the brain metastasis, although typically this was confined to 110% of the prescribed dose. All dose calculations were performed with Eclipse v. 8.6.07 using the AAA calculation model with a calculation grid of 2.5 mm.

### VMAT quality assurance

The quality of the VMAT plans was assessed according to the following measures:

Dose homogeneity using MDPD, defined as the maximum dose (MD) divided by the prescription dose (PD). This dosimetric parameter for homogeneity was defined by the RTOG in SRS Quality Assurance (QA) guideline [19]. We used the same criteria from the RTOG guidelines, that is, MDPD should be <1.25. An MDPD between 1.25 and 1.40 constitutes a minor variation and an MDPD >1.40 is a major deviation.

Dose conformity for the target metastases was quantified using PITV, defined as the prescription isodose volume (PI) divided by the target volume (TV). In the RTOG QA guidelines [19], PITV should be kept as close to 1.0 as possible while maintaining target coverage and target homogeneity criteria. A PITV between 1.0 and 2.0 is optimal. A PITV between 2.0 and 2.5 is a minor variation and a PITV >2.5 is a major deviation. Routine department quality assurance (QA) was performed by Physics for each plan through a method of film dosimetry. For QA for each plan, film was placed in a cylindrical phantom and irradiated. Planned arcs were copied onto a CT data set of the cylindrical phantom, calculated, and a dose plane equivalent to the film’s area was exported. Films were scanned and compared to the exported planer dose from the planning system. Using an in-house film dosimetry analysis program, plans were judged suitable if the plan versus film analysis passed a 3mm/ 3% gamma-value analysis test [20].

## Results

Thirty patients with 73 brain metastases were treated between March 2010 and February 2012 with VMAT. Patient demographics are summarized in Table 1. The median patient age at diagnosis of the treated brain lesions was 60 years (range: 26–84 years). The male to female ratio was 2:1. For 26 patients, BMs arose from primary melanoma and for the remaining four patients from non-small cell lung cancer (n= 2), primary breast cancer, and sarcoma. The median number of lesions per patient at the time of radiotherapy treatment was 2 (range: 1–8).

**Table 1:** Patient characteristics

	**Value**	**N**
Patient Sex	Male	20
	Female	10
Age at Diagnosis	Median (Range)	60 (26–84)
Indication	Melanoma	26
	Others*	4
Treatment	WBRTHA*	1
	WBRTHA SIB*	17
	WBRT SIB	5
	SIBHA	2
	SIB Only	5

others*: lung (2), breast (1) and sarcoma (1), WBRTHA*: whole brain radiotherapy with Hippocampal avoidance, SIB*: simultaneous integrated boost.

The anatomical locations of the brain lesions are summarized in Table 2. The majority of the lesions were located in the frontal lobe (33%). The median follow-up after completion of radiotherapy for 22 patients was 3.5 months (range: 0.03–16.5 months); seven patients were lost to follow-up, and one patient had treatment planning only before deteriorating from extracranial disease. At last-follow-up of the cohort, 11 patients were deceased, including the patient who had only treatment planning.

**Table 2:** Lesion location

**Location**	**No. of lesions**	**%**
Frontal	24	33%
Parietal	14	19%
Temporal	9	12%
Occipital	7	10%
Cerebellar	6	8%
Brain stem	3	4%
Other*	10	14%
**Total**	**73**	100%

Other*: Ventricular, thalamus, parieto-occipital, orbital, centrum, pons, midbrain, pre-central gyrus, inter-hemispheric fissure.

**Figure 1:**
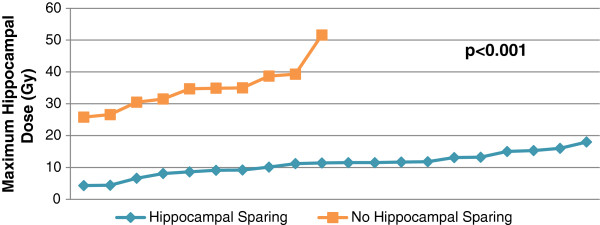
Maximum hippocampal dose for patients having HA versus no HA p<0.001.

**Figure 2:**
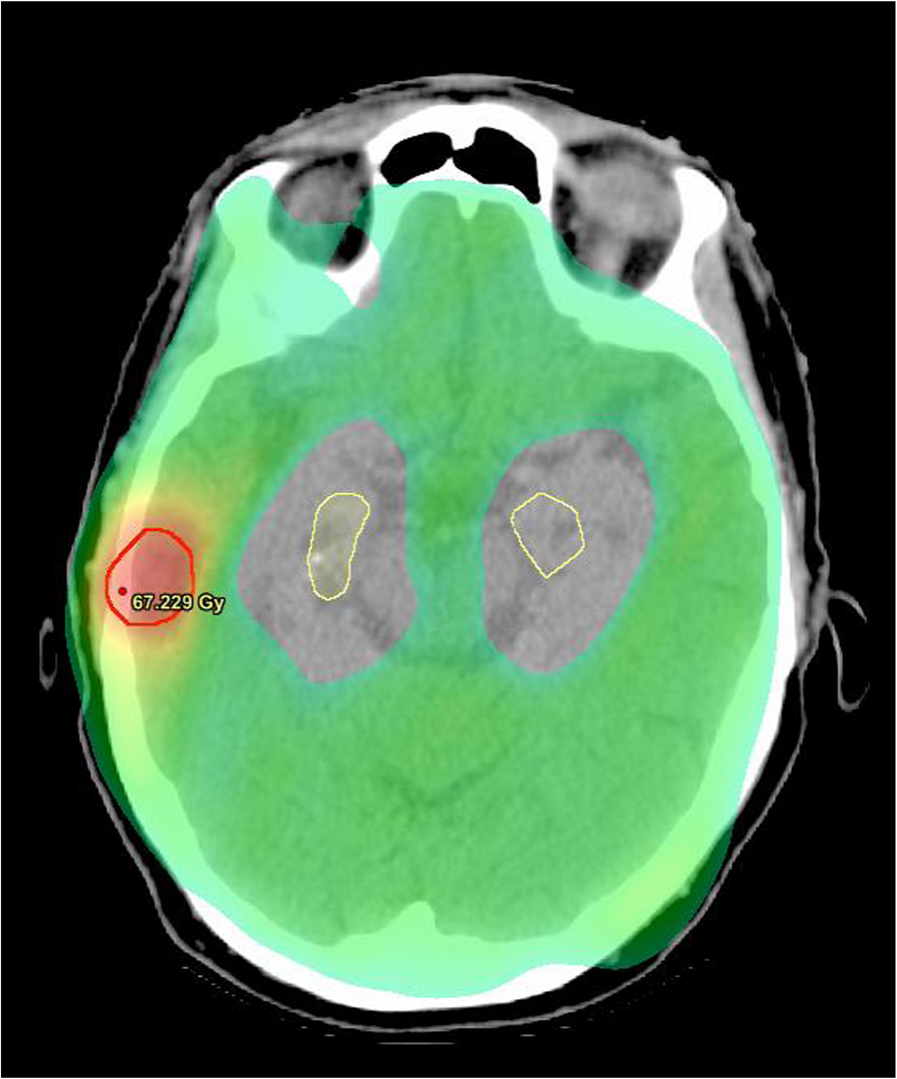
**VMAT dose colour wash for WB_ SIB_HA on planning CT of a representative patient.** Dose to metastasis was escalated (red, 63 Gy), WB at lower dose (green, 32.5 Gy), with HA (contour yellow).

Priority was placed on giving sufficient SIB dose, and HA was not attempted in these cases where lesions where close to the hippocampi, usually within 10mm. Twenty out of twenty two of the patients treated with WBRT had WBRTHA. The mean hippocampal dose for these patients ranged from 4.3 to 18.0 Gy and the maximum dose ranged from 8.4 to 32.2 Gy. For patients not receiving HA (n=10), the mean hippocampal dose ranged from 25.8 to 51.6 Gy and the maximum dose ranged from 24.3 to 56.5 Gy (Figure 1). The maximum and mean hippocampal dose were each significantly increased for patients not receiving HA (p<0.001, Mann–Whitney U). Figure 2 shows dose distribution in the WB and BMs with HA on an axial planning CT slice from a representative patient.

Twenty-two patients received WBRT with SIB to metastatic lesions, one patient received WBRTHA only, and seven patients received high dose fractionated VMAT only as listed in Table 3. WBRT was conducted for 23 patients with a median dose of 30 Gy (range 28.6–37.5 Gy). The median dose to BMs was 50 Gy (range: 20–70.8 Gy), delivered in a median of 15 fractions. Median beam-on treatment time recorded for 28 patients was 3.43 minutes (range: 1.2–6.3 minutes).

**Table 3:** Planning summary for 73 lesions (30 patients)

**Specification**	**Abbrev.**	**Units**	No*	**Median**	**Mean**	**Minimum**	**Maximum**
Whole-brain radiotherapy (Gy)*	WBRT	pts*	23	30.0	30.6	28.6	37.5
Fraction	Frx	les	73	10.0	11.8	5.0	15.0
Dose to hippocampus with HA ) (Gy)	HA	pt	20	21.9	20.4	8.4	32.2
without HA (Gy)	-	pts	10	29.0	41.1	24.3	56.5
Simultaneous integrated boost (Gy)	SIB	pts	29	45.0	44.8	20.0	70.8
Gross target volume (cc)*	GTV	les*	71	0.6	6.9	0.02	291.3
Planning target volume (cc)	PTV	les	67	3.3	13.3	0.4	344.0
Prescription isodose volume (cc)	PIV	les	64	2.4	12.6	0.1	389.0
Maximum dose (Gy)*	MD	les	73	45.6	47.1	22.0	72.0
Prescription dose (Gy)	PD	les	71	45.0	44.8	20.0	70.8
Conformity index	PITV	les	64	3.2	8.6	0.2	70.0
Homogeneity	MDPD	les	71	1.06	1.06	1.01	1.10
Beam time (min)	BT	les	68	3.4	3.6	1.2	6.3

No*: number, (Gy)*: Graypts*: patient, les*: lesion, (cc)*: cubic centimetre, (Gy)*: Gray.

As shown in Table 3, MDPD was in the optimal range (<1.25) for all lesions. The PITV was in the optimal range for 20% of lesions, with minor and major deviations in 9% and 59%, respectively, in measurable lesions (n=64). Eighty-two percent of lesions having major deviations from optimal PITV (n=38) had GTV less than 1 cc.

### Treatment outcomes

Tumour control was able to be assessed for 27 lesions (37%) in 15 of the 29 patients who had SIB (excluding the 1 patient who had adjuvant WBRTHA) at the time of the analysis. Twenty two lesions (81%) were controlled (any response or stable disease) for 13 patients, and five lesions (29%) were uncontrolled in four patients at a median follow up of 3.5 months. Three patients showed distant intracranial failure yet with controlled treated BMs. For the controlled (22) versus uncontrolled (5) lesions, there was no significant difference in median GTV, PTV, prescription dose (PD), SIB dose, WBRT dose, PITV. Four patients (7 lesions) died before assessment of tumour control was done. For the remaining 39 lesions (13 patients), it was not possible to assess treated disease control at data collection time in this retrospective cohort.

Overall survival from the end of radiotherapy for 22 patients was a median of 9.40 months (95% CI: 1.95–16.85). HA, number of lesions, patient age and sex, WBRT dose and SIB dose did not significantly influence overall survival.

Acute toxicity was minimal, and was predominantly alopecia, headache and vomiting. Grade I was recorded for 5 patients, grade I/II for 2 patients, and grade II for 3 patients. One patient experienced grade IV late toxicity. This was histologically proven brain necrosis without tumour recurrence in two melanoma lesions following ipiluminab, a phenomenon which has been reported in other studies [21].

## Discussion

The main goal of palliation is to improve quality of life and perhaps increase quantity of life. Important quality of life endpoints for patients suffering with metastatic melanoma to the brain include control of neurological symptom and with as less impact on NCF as possible. VMAT with WBRTHA and SIB may deliver all these in one radiotherapy course. This study reports the experience of using VMAT to streamline WBRT with hippocampal sparing and SIB in the Australian context.

Our study compromised 30 patients with 73 brain lesions. The 26 patients with 54 lesions were from primary melanoma. For 20 patients treated with HA, the mean hippocampal dose ranged from 4.3 to 18.0 Gy and the maximum dose ranged from 8.4 to 32.2 Gy, compared to mean hippocampal dose ranged from 25.8 to 51.6 Gy and the maximum dose ranged from 24.3 to 56.5 Gy for patients not receiving HA (n=10). The median treatment time per fraction, recorded for 28 patients, was 3.43 minutes which compared to WBRT time of 1.3 minutes. This compares well with Hsu et al. [22]. This treatment therefore saves machine time when compared even to other conformal techniques, as has been found in other tumour sites [15]. More importantly, VMAT minimises time spent at hospital with the single planning session and short daily fraction time.

For the patients treated with SIB, our VMAT plans met the RTOG SRS guidelines. MDPD was in the optimal range (<1.25) for all lesions, while the PITV was in the optimal range for 20% of lesions, with minor and major deviations in 9% and 59% respectively in measurable lesions (n=64). This high percentage of major PITV deviation might explained by the fact that 82% of lesions (n=38) having major deviations from optimal PITV had GTV less than 1 cc. PITV does not appear to be important for lesions < 1 cc [23].

In our study, twenty two of twenty-seven (81%) assessable lesions in 22 melanoma patients were controlled at a median time of 3.5 months after completion of radiotherapy. This compares well with the reported results of SRS in melanoma BMs of other studies [24-26]. There was distant intracranial failure in 2 patients, yet with controlled metastases which is comparable to the control seen in other studies of WBRT in melanoma [27]. Our results show that VMAT can deliver comparable results to the current conventional treatments of SRS with or without WBRT. Shehata et al. [28] reported local control rate of 97% versus 87 %,for 468 BMs treated SRS ± WBRT, respectively, including melanoma patients. Other series of Gamma Knife SRS alone in melanoma patients with BMs showed a local control rate of 83% [29].

Acute toxicity overall was minimal, only one patient experienced grade IV late toxicity. These results are similar to the findings of Weber et al. [30] reported the achievement of delivery of WB VMAT 30 Gy and a SIB to the BMs 40 Gy in 10 fractions with no significant toxicity and stable Qol during treatment. Formal pre and post treatment quality of life (Qol) assessment was beyond the scope of this retrospective analysis.

We report an overall survival of 9.4 months. In a multi-institutional retrospective review [31] of almost 4000 patients with newly diagnosed BMs, the median survival was 6.74 months for 481 metastatic melanoma patients treated with SRS ± WBRT. A pooled analysis [32] of 120 patients treated with VMAT SIB for dose-escalation of oligometastatic disease of the brain, reported a median overall survival of 5.9 months after a median follow up of 4.7 months for the entire group. There were no cases of patient death attributable to toxicity from the SIB brain radiotherapy. VMAT is at least comparable to current treatment techniques in terms of overall survival.

This report shows that VMAT can deliver effective and efficient WBRT with either HA, SIB, or both in patients with BMs. The ability to do this is becoming more important as melanoma patients are living longer with new targeted therapies [33] that can even cross the blood brain barrier and have efficacy in the brain. The resulting increase in progression free survival means avoiding WBRT toxicity is even more important.

## Conclusion

This is the first reported Australian experience of patients treated with WBRT with either HA, SIB, or both via VMAT. This treatment is feasible and tolerable. VMAT was able to deliver SRS quality dose distributions to individual metastases while adequately delivering WBRT and conformably sparing the hippocampus. The mean time to deliver this treatment with VMAT was less than 3.43 min, making this treatment approach attractive from the practical standpoint. There was no additional toxicity and survival was similar to conventional treatments.

## Consent

Written informed consent was obtained from all patients for publication of this report and any accompanying images.

## Competing interest

The authors declare that they have no competing interests.

## Authors’ contributions

GF and RA coordinated the entire study. Data collection was conducted by RA, GF, AH, PK and DN. Data were analyzed by LH, RA, GF and AH. All authors read and approved the final manuscript.
